# High performances of dual network PVA hydrogel modified by PVP using borax as the structure-forming accelerator

**DOI:** 10.1080/15685551.2017.1382433

**Published:** 2017-09-29

**Authors:** Min Huang, Yi Hou, Yubao Li, Danqing Wang, Li Zhang

**Affiliations:** ^a^ Research Center for Nano-biomaterials, Analytical & Testing Center, Sichuan University, Chengdu, China; ^b^ Department of Obstetrics and Gynecology, West China Second University Hospital, Sichuan University, Chengdu, China

**Keywords:** Hydrogel, cross-linking, borax, polyvinylalcohol, polyvinylpyrrolidone

## Abstract

A dual network hydrogel made up of polyvinylalcohol (PVA) crosslinked by borax and polyvinylpyrrolidone (PVP) was prepared by means of freezing-thawing circles. Here PVP was incorporated by linking with PVA to form a network structure, while the introduction of borax played the role of crosslinking PVA chains to accelerate the formation of a dual network structure in PVA/PVP composite hydrogel, thus endowing the hydrogel with high mechanical properties. The effects of both PVP and borax on the hydrogels were evaluated by comparing the two systems of PVA/PVP/borax and PVA/borax hydrogels. In the former system, adding 4.0% PVP not only increased the water content and the storage modulus but also enhanced the mechanical strength of the final hydrogel. But an overdose of PVP just as more than 4.0% tended to undermine the structure of hydrogels, and thus deteriorated hydrogels’ properties because of the weakened secondary interaction between PVP and PVA. Likewise, increasing borax could promote the gel crosslinking degree, thus making gels show a decrease in water content and swelling ratio, meanwhile shrinking the pores inside the hydrogels and finally enhancing the mechanical strength of hydrogels prominently. The developed hydrogel with high performances holds great potential for applications in biomedical and industrial fields.

## 1. Introduction

Hydrogels are cross-linked network structure of water soluble polymer or hydrophilic polymer, which can swell in water to capture many times of their original mass [1]. Nowadays, interests in developing polymer hydrogels continue to grow because of their excellent hydrophilicity, permeability and viscoelasticity, which raise people’s high expectations for hydrogels with high performances. Hydrogels have been applied in biological, pharmaceutical, and daily-care applications, such as immunoassay, vehicles for drug delivery, eye contact lenses, implantable artificial muscles, and wound dressings, etc. [2 3 4 5 6 7 8 9 10 11 12]. However, hydrogels made of single polymers usually display poor mechanical behaviors [13], as we know, single polyvinylalcohol (PVA) hydrogels are hard to meet the requirements of some applications needing high mechanical strengths. Many efforts have been made to improve the mechanical properties of hydrogels, of which a dual-network has been typically used [14 15 16].

PVA, made of polyvinyl acetate, is a kind of good biocompatible, non-toxic and non-carcinogenic water-soluble polymer. Due to the hydrophilic functional groups in each molecular unit, PVA can form chemically and physically into a crosslinked hydrogel [17]. Usually at the cost of its stability and mechanical properties, PVA can be easily fabricated into PVA hydrogel by absorbing plenty of water [18]. PVA is very sensitive to borax, and introducing borax as a crosslinker into PVA hydrogel could significantly enhance the malleability but the mechanical property and ductility were still poor [19]. The PVA–borax system also exhibited non-Newtonian behavior, leading to flow under low stress and limited dimensional stability [19]. To overcome this, polyvinylpyrrolidone (PVP) was added to the PVA–borax system to stabilize the network by forming the inter-chain hydrogen bonding [20]. PVP has planar and highly polar side groups owing to the bond of ‘
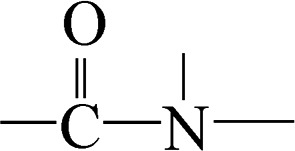
’ in the lactam ring [21] and has been used in many biomedical applications due to its remarkable properties, such as high hydrophilicity, biocompatibility and no-toxicity [22 23]. Additionally, the interactions and entanglements between PVP and PVA can also alter the morphology, swelling property, water retention and mechanical strength of the final hydrogel. As we all know, applying physical crosslinking to make PVA hydrogel is very popular because of its unique superiority compared to other crosslinking methods. For example, controlling the crosslinking conditions can not only adjust the mechanical properties of PVA hydrogel but also maintain the similar features to biological tissues [24].

Herein, we used the dual physical crosslinking method to prepare PVA/PVP composite hydrogel through using borax as a physical crosslinker first followed by freezing-thawing circles. The physio-chemical properties would be systematically investigated by varying the feeding ratio of PVA/borax and the content of PVP in order to evaluate how both borax and PVP to influence the fundamental behaviors of PVA/borax hydrogels, thus providing some valuable data and theoretical support for the further study of PVA-based hydrogels.

## 2. Experimental

### 2.1. Materials and methods

**Figure 1.**
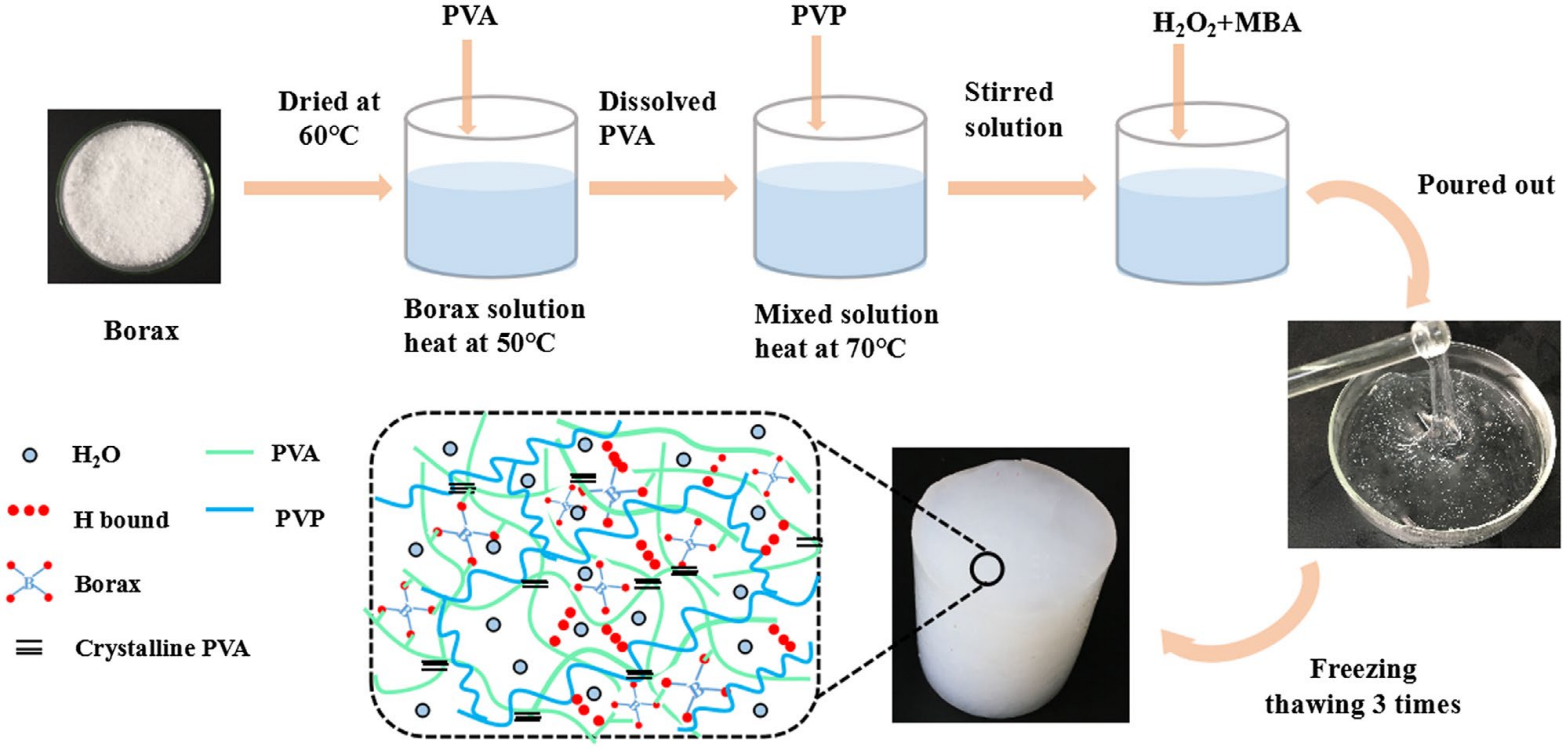
Schematic illustration of the process for preparing PVA/PVP/borax composite hydrogel.

PVA (a degree of hydrolysis ≧ 90.5% with a polymerization degree 1799 ± 50), 30 wt% hydrogen peroxide and anhydrous borax were purchased from Chengdu Kelong Co., China. 1-Vinyl-2-pyrrolidone (VP) and N,N′-methylenebisacrylamide (MBA) were supplied by Shanghai Aladdin Co., China. All these reagents were analytical grade, and were used without further purification. Firstly, borax was added to deionized water with stirring at 50 °C for 5 min till dissolved thoroughly. Then, PVA particles were put into the stirring borax solution and meanwhile heated up to 95 °C and kept stirring for 2 h to get a 10 wt% PVA solution. After that, VP with the ratio of VP/(PVA + H_2_O) varying from 2 to 8% was poured into the mixture solution at 70 °C accompanied by adding 0.3 g H_2_O_2_ to initiate the free radical polymerization. After 1.5 h reaction, MBA, 1 wt% of the amount of VP, was introduced to crosslink PVP with stirring for another 30 min to obtain a composite gel. Subsequently, the transparent gel was cast into the mold and cooled down to room temperature, and then was frozen under −20 °C for 12 h and thawed for another 12 h under room temperature. The freezing-thawing circle was repeated 3 times and finally obtained the sample. The whole process for making PVA composite hydrogel was schemed in Figure 1.

### 2.2. Characterization

#### 2.2.1. Water content

Water on the sample’s surface was removed with filter paper before weighing, and then the sample was freeze-dried to constant weight. The water content (*W*_*C*_) of the hydrogels was calculated based on Equation (1).

(1) $$W_{C}\;\left(\%\right)=\frac{W_{1}-W_{2}}{W_{1}}\times 100\%$$

where *W*_1_ and *W*_2_ are the respective weight of the hydrogel before and after being freeze-dried. Each weight was measured three times to get an average value.

#### 2.2.2. Swelling ratio

To investigate the swelling ratio (*W*_*S*_) of the hydrogels, the freeze-dried sample was immersed into the deionized water under 30 °C until a swelling equilibrium. Likewise, the surface water was wiped off before measuring the sample’s weight. The swelling ratio (*W*_*S*_) was calculated by Equation (2).

(2) $$W_{S}\;\left(\%\right)=\frac{W_{3}-W_{2}}{W_{2}}\times 100\%$$

where *W*_2_ is the sample weight in dry state, and *W*_3_ is the sample weight in swollen-saturated state. Each testing was repeated three times to get an average value.

#### 2.2.3. FT-IR characterization

FT-IR spectra of PVP, PVA/borax and PVA/PVP/borax composite hydrogels were evaluated by infrared spectrometer (Nicolet 6700, American), with a wave number ranging from 670 to 4000 cm^−1^.

#### 2.2.4. Morphology observation

The morphology of the freeze-dried hydrogel samples were observed by scanning electron microscopy (SEM, JSM-6510LV) using 10 kV for secondary electron imaging after being gold-coated.

#### 2.2.5. Rheological analysis

Rheological tests were performed using dynamic rheometer (TA Discovery HR-2R) with the angular frequency (*ω*) in the range of 0.1–100 rad/s and the stain of 1.0%. The measuring gap size was set as 0.5 mm under 37 °C.

#### 2.2.6. Differential scanning calorimetric measurement

Differential scanning calorimetric (DSC) measurements of dried hydrogel sample was conducted using a DSC1/700 METTLER under nitrogen environment. The sample was initially cooled down to −20 °C and then heated up to 260 °C at the speed of 10 °C/min.

#### 2.2.7. Mechanical testing

Uniaxial compressive strength was tested on the universal testing machine (AGS-X, Japan) under a strain rate of 5 mm/min until the deformation reached to 80%, which was based on the testing standard for rubbers due to the similarities of hydrogels and rubbers.

## 3. Results and discussion

### 3.1. Water content and swelling behavior

The water content and the swelling degree of hydrogels with different compositional ratios were listed in Table 1. As can be seen that the water content in PVA/borax hydrogels without PVP varied from 68 to 80%, while increased obviously with the addition of PVP. This was mainly due to the oxygen atoms of the carbonyl group in PVP could form hydrogen bonds with the H atoms in water molecules, which endowed PVP with strong hydrophilicity to bond with plenty of water. But when the amount of PVP reached 4.0%, its steric hindrance became prominent and reduced the space to hold more water molecules; meanwhile, increasing PVP not only made more hydrogen bonds but also generated more entanglement points between PVA and PVP, and such dual effects were embodied by a much denser network in hydrogel, just as the crosslinking effect [25]. The denser network structure of PVA/PVP/borax hydrogel and restricted segment activity between crosslinking points hindered water from penetrating into the network, finally resulting in a reduction both in the water content and the swelling ratio [25].

**Table 1.** Water content and swelling degree of hydrogels with different compositions.

Hydrogels with different compositional ratios		Water content ± standard error (%)	Swelling degree ± standard error (%)
PVA/borax	PVP content (wt %)		
PVA/borax	PVP content (wt %)		
200:1	0.0	80.54 ± 0.05	110.54 ± 0.07
200:1	2.0	84.04 ± 0.04	137.71 ± 0.01
200:1	4.0	89.38 ± 0.07	157.26 ± 0.09
200:1	6.0	88.30 ± 0.03	156.49 ± 0.03
200:1	8.0	87 .23 ± 0.06	145.15 ± 0.08
100:1	0.0	75.38 ± 0.08	102.41 ± 0.09
100:1	2.0	86.59 ± 0.09	139.49 ± 0.04
100:1	4.0	85.51 ± 0.11	136.09 ± 0.07
100:1	6.0	83.30 ± 0.14	135.19 ± 0.18
100:1	8.0	82.23 ± 0.03	127.06 ± 0.06
66.7:1	0.0	68.15 ± 0.04	90.52 ± 0.01
66.7:1	2.0	74.27 ± 0.06	98.35 ± 0.02
66.7:1	4.0	82.29 ± 0.08	109.87 ± 0.07
66.7:1	6.0	82.15 ± 0.07	110.80 ± 0.08
66.7:1	8.0	80.01 ± 0.07	104.46 ± 0.02

As for PVA/borax hydrogels, increasing borax tended to produce more crosslinking sites, compacting the network in hydrogels to a large extent [24]. Therefore, with the PVA/borax ratio declining, the network structure of PVA hydrogel became denser and the free space to hold water went down obviously, which was exactly reflected by the lowered swelling ratio and water content from 110.54 to 80.54% for the hydrogel with PVA/borax of 200:1 to 90.52 and 68.15% for the hydrogel with PVA/borax of 66.7:1, respectively.

### 3.2. FT-IR analysis

**Figure 2.**
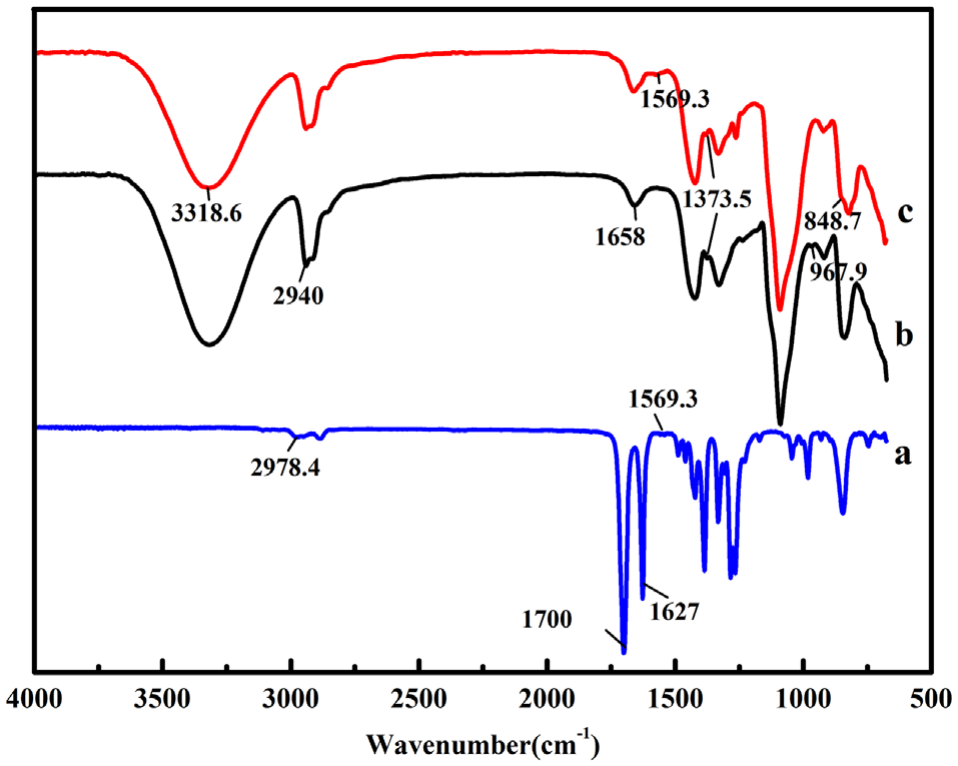
FT-IR spectra for dried gels made of PVP (a), PVA/borax with a ratio of 100:1 (b) and PVA/PVP/borax with the PVA/borax ratio of 100:1 and 4.0% PVP (c).

FT-IR spectra for hydrogels of PVP, PVA/borax and PVA/PVP/borax were presented in Figure 2. The peaks at 2978.4, 1700, 1569.3 cm^−1^ in Figure 2(a) could be assigned to the stretching vibration of C–H, C=O and C–N groups, respectively. In Figure 2(c), most of the characteristic peaks owing to PVP except for the C–N peak at 1569.3 cm^−1^ were overlapped with those absorption peaks from PVA and borax. Besides, a broad band showing up at 3318.6 cm^−1^ was observed both in Figure 2(b) and (c), which belonged to O–H stretching vibration from the hydroxyl groups either complexed with borate ions or formed intermolecular hydrogen bondings [26]. The peaks at 2940 and 1373.5 cm^−1^ in both spectrum b and c were attributed to C–H stretching in CH_2_ groups and the asymmetric stretching of B–O–C, respectively [27]. It is worth noting that the absorption peaks for PVA/PVP/borax hydrogel arising at 967.9 and 848.7 cm^−1^ were assigned to the out-of-plane bending vibration of BH_2_ and B–O stretching from the residual B(OH)_4_, respectively, confirming the crosslinking between PVA and borax as well as the presence of a borax network and small amount of B(OH)_4_ within the PVA/PVP/borax hydrogels [19 27 28 29].

### 3.3. SEM observation

**Figure 3.**
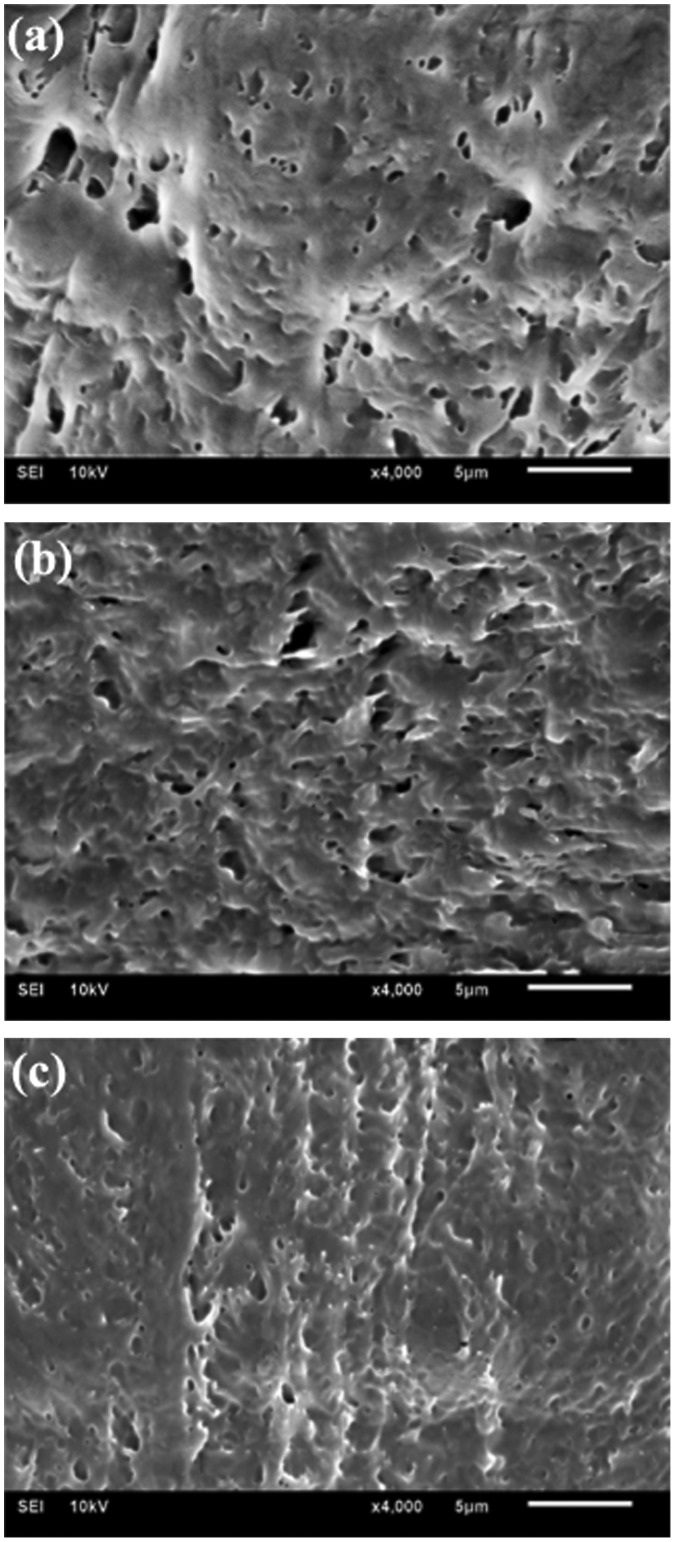
SEM images for PVA/PVP/borax hydrogels with changing PVA/borax ratio: 200:1 (a), 100:1 (b) and 66.7:1 (c). The content of PVP in hydrogels was set as the constant of 2.0%.

The influence of borax content on the network structure of hydrogels was characterized by SEM, as shown in Figure 3. It was obvious that all the freeze-dried samples showed 3-D porous structures with the removal of water from the hydrogels. Nevertheless, the morphologies were different from each other. For example, the hydrogel with a higher PVA/borax ratio of 200:1 displayed irregular and unevenly distributed pores inside (Figure 3(a)); while the hydrogel with a medium PVA/borax ratio of 100:1 demonstrated smaller pores and a denser structure together with a better arrangement of molecular chains compared to the former (Figure 3(b)); it was worthy of pointing out that lowering the PVA/borax ratio to 66.7:1 (Figure 3(c)) resulted in a much denser network structure, and the arrangement of molecular chains became even more orderly, indicating that the existence of borax played the role of compacting molecular chains together and made more entanglements, finally caused a decreased water content. It had also been reported that the increase in borax content led to a promotion in the crosslinking degree of hydrogel, resulting in a denser and more compact structure [19]. Moreover, the structure of hydrogels was correlated with the solution viscosity [30]. Thus, the more borax was introduced, the more viscous the PVA solution was. After several freezing-thawing circles, eventually we got a final hydrogel with a denser network structure and a relatively more orderly-arrangement molecular chain, which was consistent with the changing trends of the water content and the swelling ratio.

**Figure 4.**
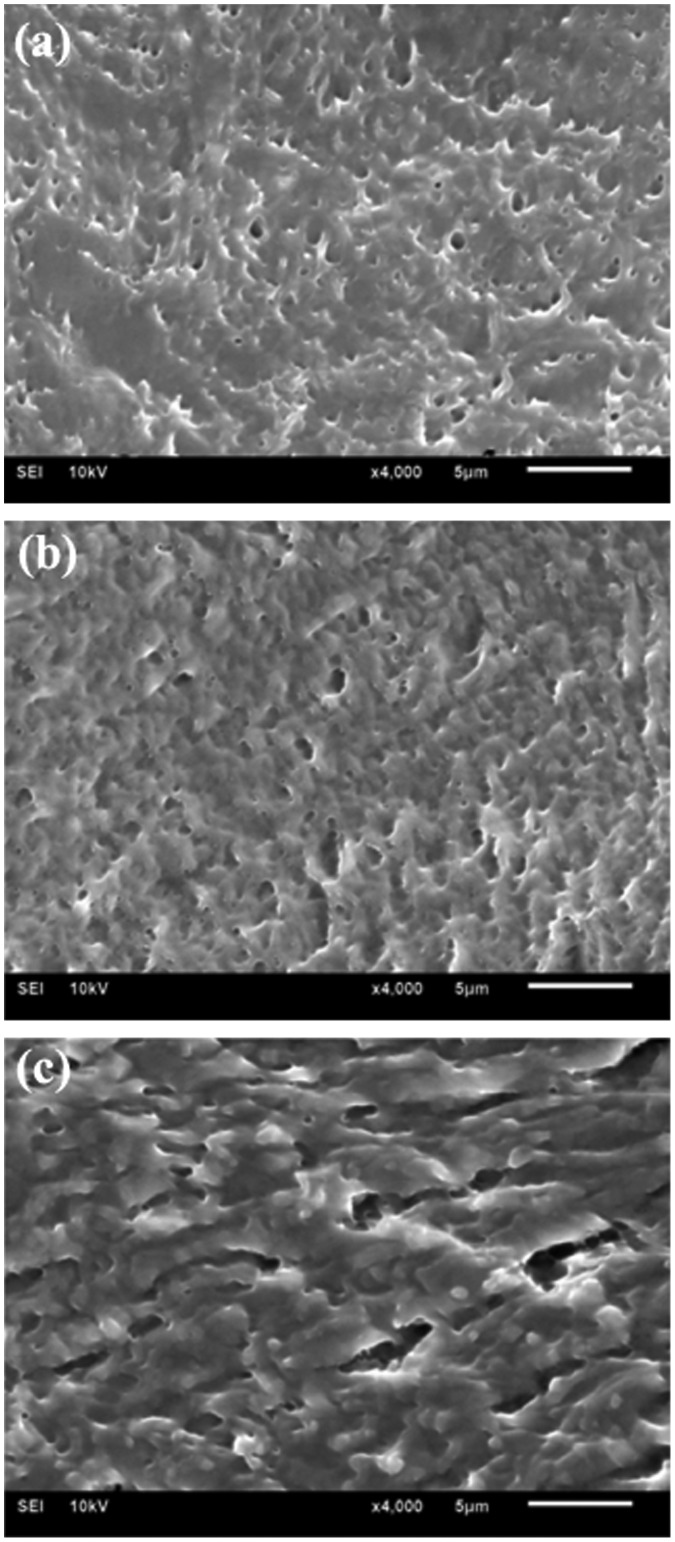
SEM images of freeze-dried hydrogels with different PVP content: without PVP (a), 4.0% PVP (b) and 8.0% PVP (c). The ratio of PVA/borax for all hydrogels was set as 100:1.

For the PVA/PVP/borax hydrogel, except for making clear of the influence of PVA/borax ratios, the effect of PVP on the final hydrogel was also necessary to be known by changing its content. Figure 4 displayed SEM images of PVA/PVP/borax hydrogels with varying PVP contents. It could be clearly seen that the pore size in hydrogels went up gradually with the increase of PVP content. In particular, the incorporation of 4.0% PVP made pore sizes more even and the distribution more uniform (Figure 4(b)). While introducing 8% PVP instead caused uneven pore size and distribution as well as an extremely non-uniform structure (Figure 4(c)), which was caused by the increasing entanglement between PVA and PVP molecular chains with PVP content increasing. Meanwhile, PVP had a high water-adsorbing capacity, during freezing macromolecular chains were squeezed together by the swollen volume of liquid water changing into solid ice which evaporated up, finally, leaving large pores [31]. Similar result was also reported by Spoljaric et al. [19].

### 3.4. Dynamic viscoelasticity

**Figure 5.**
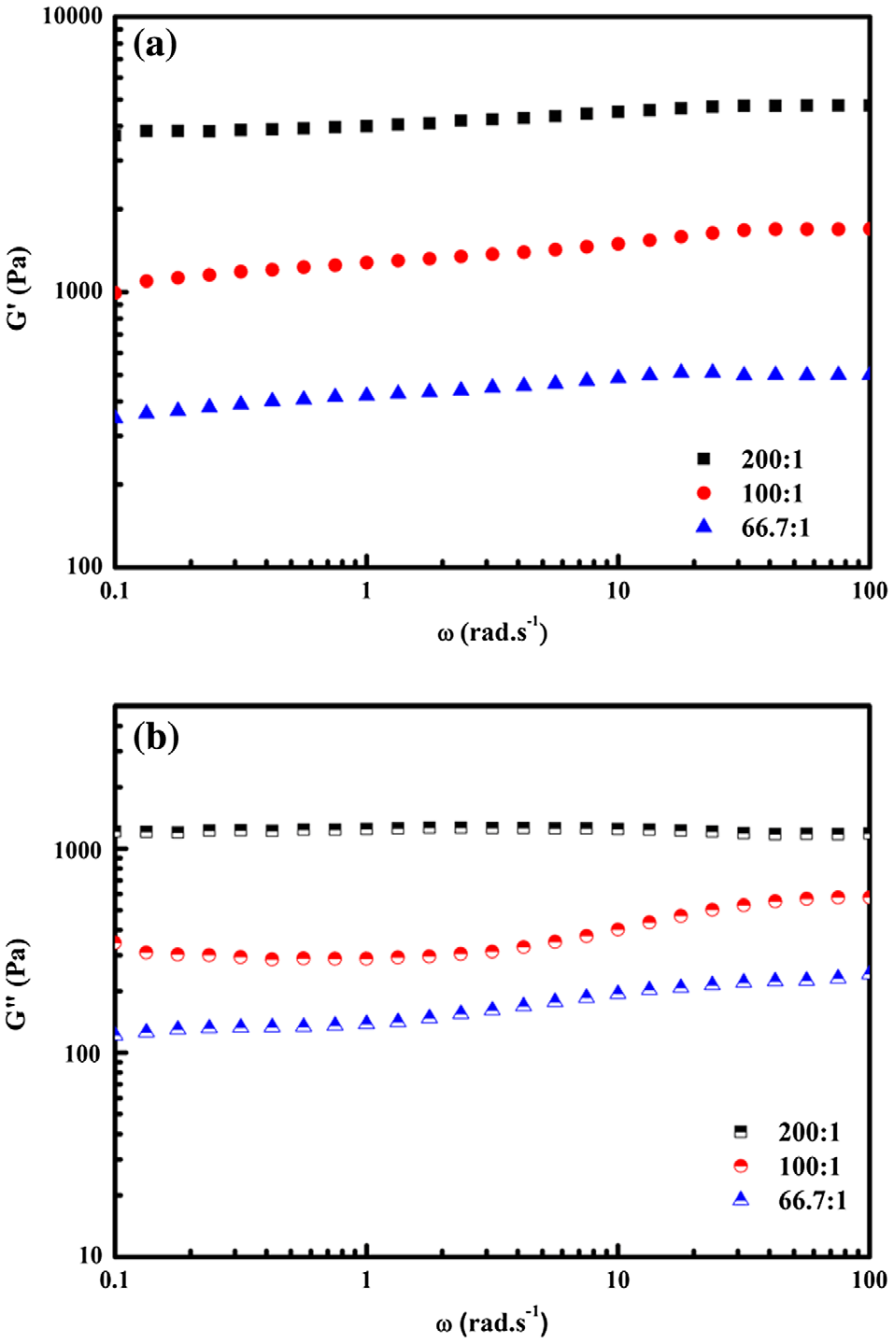
The relationship of the storage modulus G′ (a) and the loss modulus G″ (b) with angular frequency for hydrogels with different PVA/borax ratios. The content of PVP was set as 2.0%.

The mechanical properties of PVA/PVP/borax hydrogels were examined by oscillatory rheological experiments as a function of angular frequency. Figure 5 showed the changes in the storage modulus (G′) and the loss modulus (G″) for the hydrogels with different PVA/borax at a constant PVP content of 2.0%. The storage modulus (G′) is the amount of energy stored in the elastic deformation during deforming the material, reflecting the elasticity of those tested samples. And the loss modulus (G″) is the amount of energy lost due to the viscosity deformation, reflecting the viscosity of the materials [32]. As can be seen from Figure 5(a), G′ of all the samples was independent on the angular frequency, but climbed up with the increase of PVA/borax ratios. And the similar trends also happened to G″ shown in Figure 5(b).

The independence of G′ on frequency signified that the macromolecular chains could be consistent with the change of the angular frequency, and the hysteresis effect was very low [33]. While the changing trend of G′ with PVA/borax ratios indicated that reducing the amount of borax could be helpful for the high frequency motion.

**Figure 6.**
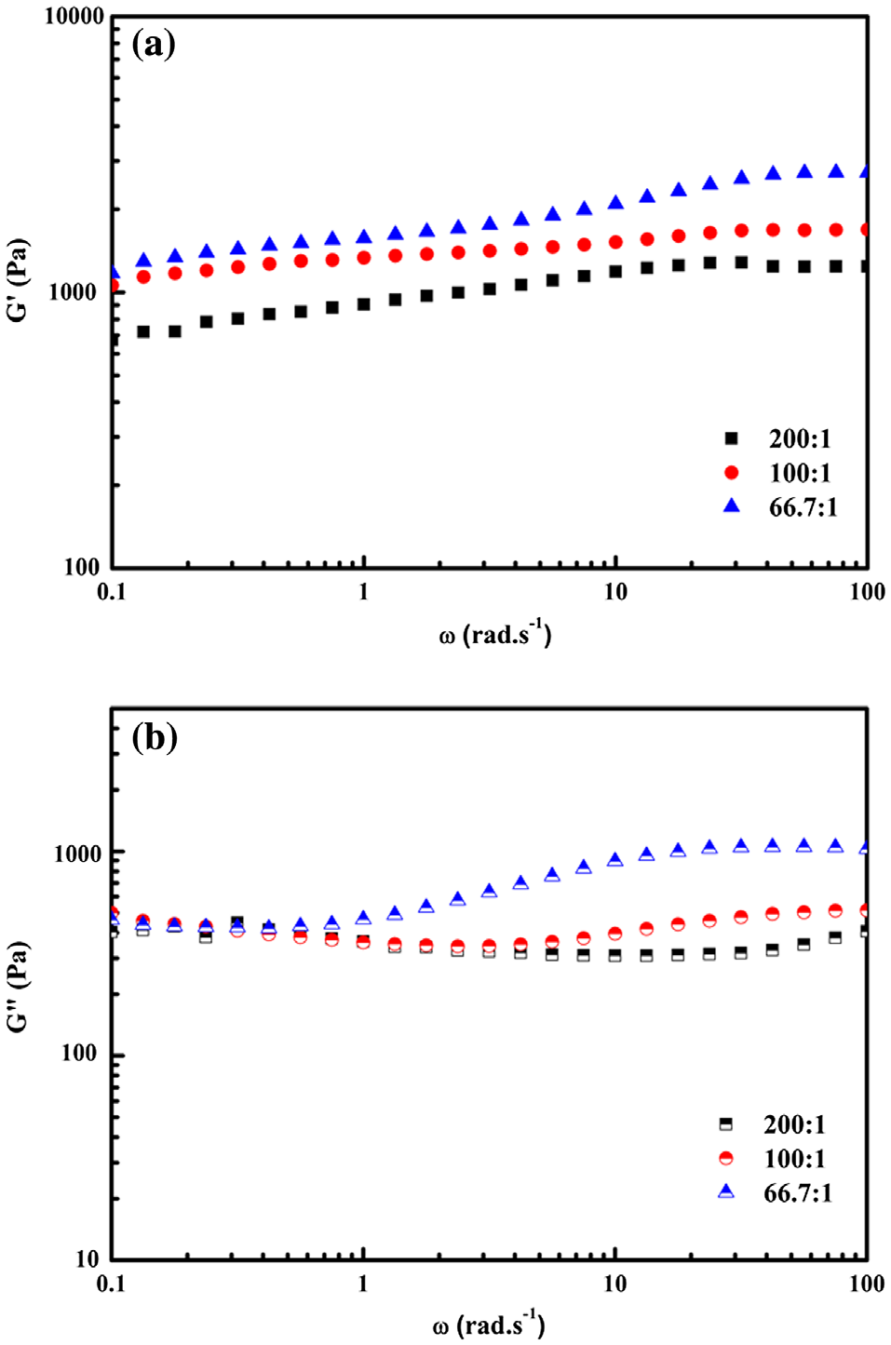
The relationship of the storage modulus G′ (a) and the loss modulus G″ (b) with angular frequency for hydrogels with different PVA/borax ratios. The content of PVP was set as 6.0%.

However, the totally opposite phenomena occurred for all hydrogels with different PVA/borax ratios at 6.0% PVP presence. Both G′ and G″ decreased with the increase of PVA/borax ratio, as shown in Figure 6. It could be explained that adding more PVP tended to barrier the interaction between the functional groups in PVA and borax, thus reduced the bulk density of hydrogels and finally made the hydrogel more elastic.

**Figure 7.**
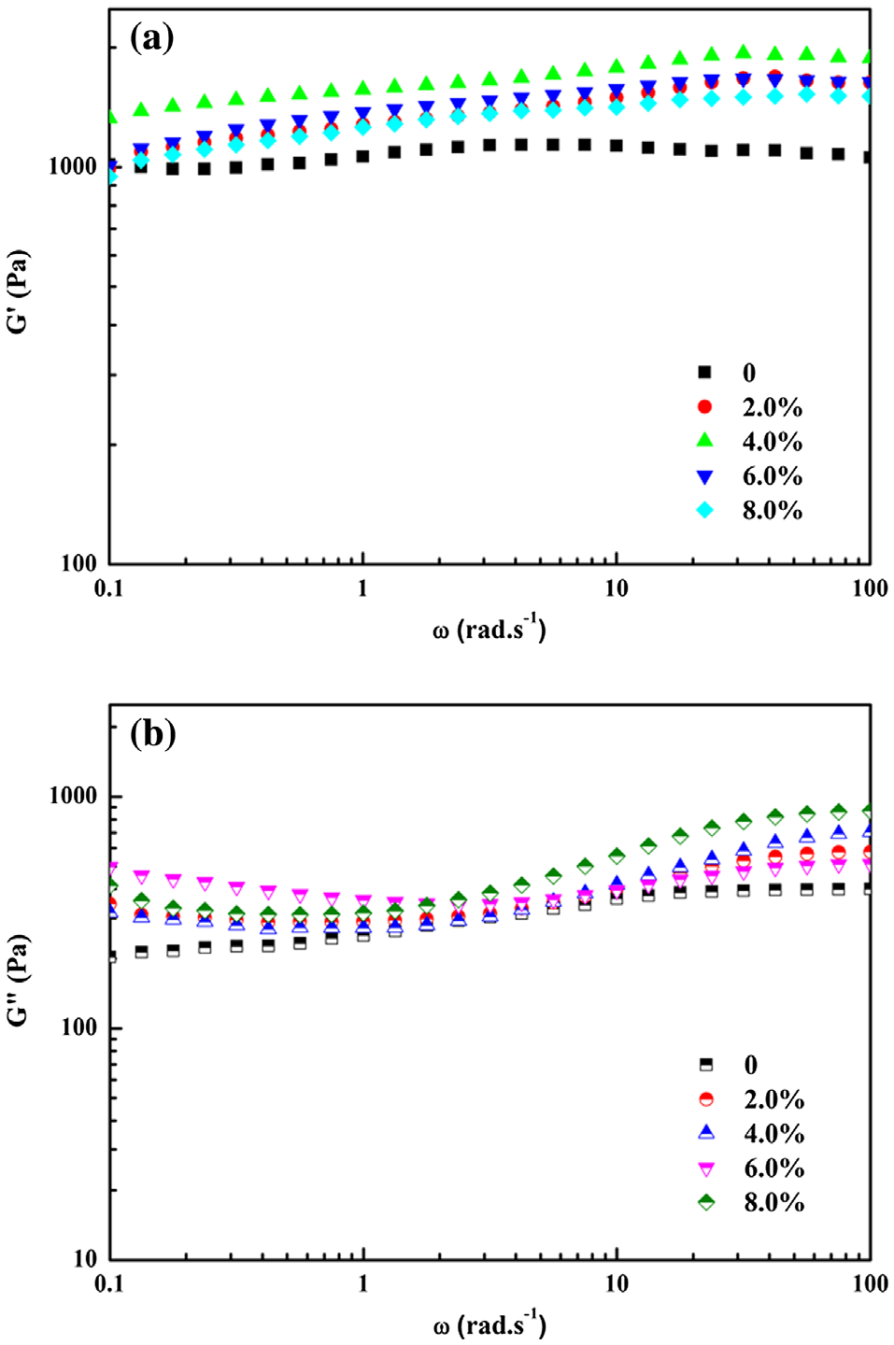
The relationship of the storage modulus G′ (a) and the loss modulus G″ (b) with angular frequency for hydrogels with changing PVP content. PVA/borax ratio was set as 100:1.

Figure 7 displayed the influence of PVP content on the storage modulus G′ and the loss modulus G″ of hydrogels with the constant PVA/borax ratio of 100:1. It was obvious that G′ for all samples slightly increased with angular frequency, but the changing tendency showed an increase with PVP content going up to 4.0% and then displayed a bit decrease with PVP content increasing continually to 6% and up, indicating a strong interaction and entanglement between PVP and PVA molecular chains at low PVP content [34]. It was worth noting that the G′ of PVA/borax hydrogel was independent on the angular frequency. Further increasing PVP content instead weakened the interaction between PVA and borax as well as between PVA and PVP, resulting in a decrease in elastic modulus, which was in line with the results discussed in Figure 6. Similar conditions also occurred in PVA/PVP gels [35].

### 3.5. Degree of crystallinity and mechanical properties

**Figure 8.**
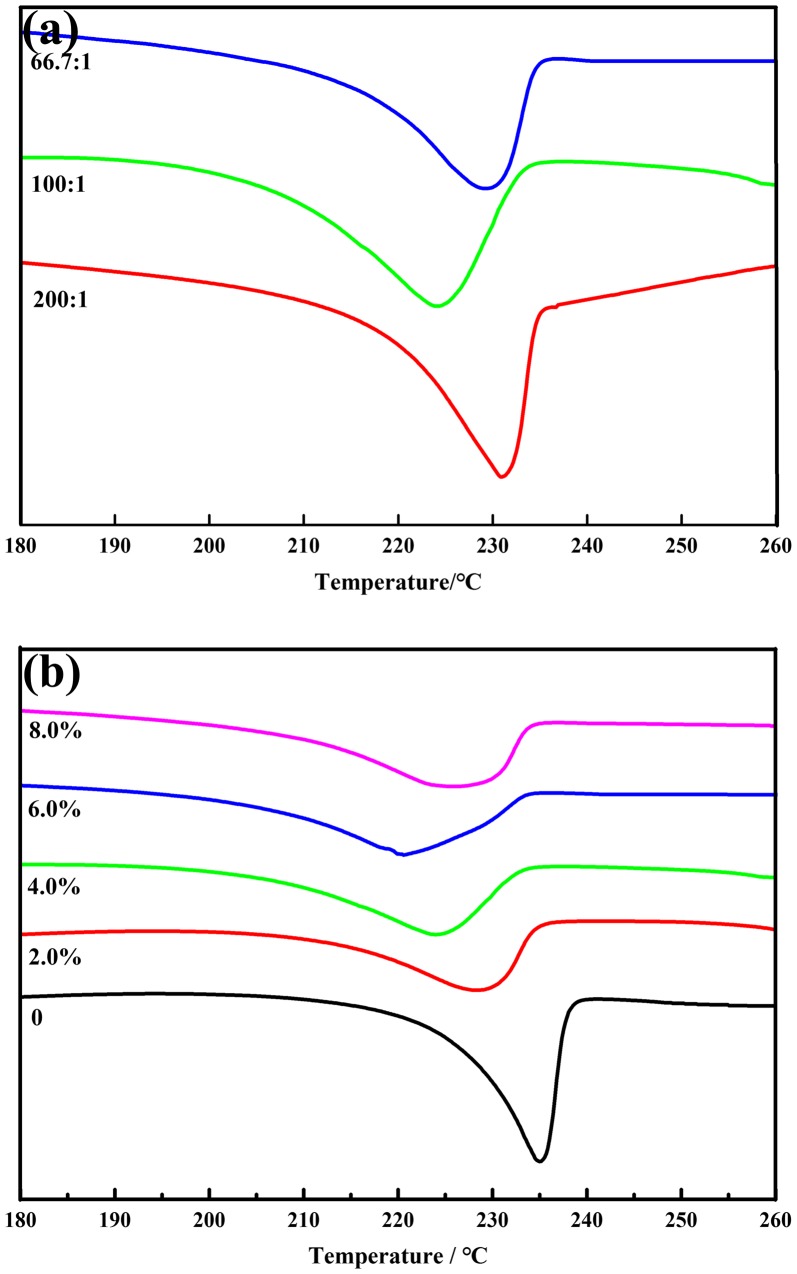
DSC thermograms of hydrogels with different PVA/borax ratio (PVP content was set as 4.0%) (a) and changeable PVP content (PVA/borax ratio was set as 100: 1) (b).

The crystallinity of PVA/PVP/borax hydrogels was proportional to its melting enthalpy, and it was calculated according to the formula *χ*_*c*_ = Δ*H*/Δ*H*_*C*_ [36], where Δ*H* represents the heat required for melting a sample, determined by integrating the area under the melting peak over the range 180–260 °C, and Δ*H*_*C*_ is the theoretical heat needed for melting a 100% crystalline PVA. For PVA, Δ*H*_*C*_ = 138.6 J/g, which was taken from literature [37]. Figure 8(a) and (b) showed the melting peak of hydrogels with variable PVA/borax ratios and changeable PVP content, respectively. The crystallinity and the compressive strength of selected samples were summarized in Table 2. As can be concluded that under the constant PVP content, the compressive strength of a hydrogel was proportional to the borax content. As discussed above, the increase in borax content enhanced the crosslinking degree of hydrogels. While the higher crosslinking degree caused the closer distance between the chain segments and made the movement of molecular chains more difficultly. When a hydrogel with higher crosslinking degree was subjected to external forces, as a result it tended to perform a larger compressive capacity.

**Table 2.** Crystallinity and compressive strength of selected hydrogels.

PVA/borax	PVP content (wt%)	*χ*_***c***_ (%)	Strength ± standard error (MPa)
100:1	0.0	58.9	0.83 ± 0.01
100:1	2.0	40.4	1.23 ± 0.02
100:1	4.0	46.0	1.61 ± 0.01
100:1	6.0	50.5	0.85 ± 0.03
100:1	8.0	55.3	0.48 ± 0.01
200:1	4.0	50.6	1.45 ± 0.02
66.7:1	4.0	58.6	1.82 ± 0.01

The calculated crystallinity (*χ*_*c*_) of PVA/borax hydrogel was 58.9%, while it decreased to 40.4% by adding 2% PVP, which could be explained that the molecular chains of PVP inserted into and separated PVA macromolecules, thus impeded the process of PVA crystallization [38]. While increasing PVP content continuously, the crystallinity began to increase instead. We speculated that high PVP content increased the numbers of bonds between PVP and PVA/borax network, which limited the movement of molecular chains to a certain extent, thus increased the crystallinity of hydrogels. If only considering the crystallinity, PVA/borax hydrogel should have higher mechanical strength than PVA/PVP/borax hydrogel, because the addition of PVP partly destroyed the PVA/borax network and the interaction between PVA molecular chains. Similar situation had happened to PVA/PVP hydrogel reported by Gong et al. [38]. However, when PVA/PVP/borax system went through several freezing-thawing circles, a well miscible network structure could be formed at molecular level by the newly formed intermolecular hydrogen bonds between the three compositions, contributing a lot to the mechanical properties. It was noteworthy that when PVP content went up over 4%, the compressive strength of hydrogels dropped slightly, because the secondary interaction between PVP and PVA chains was weakened by adding overdosed PVP, indicating excessive PVP would bring adverse influence to the mechanical properties of hydrogels [35].

## 4. Conclusion

In this work, PVP/PVA/borax hydrogels with different compositional ratio were prepared by the freezing-thawing technique. The effects of PVP and borax on the hydrogels were evaluated by comparing the physical and chemical properties of the two hydrogels made of PVA/borax and PVA/PVP/borax. Increasing the borax content in a reasonable range resulted in a more compact structure, exhibiting lower water content and high compressive strength. PVA/PVP/borax hydrogel with 4% PVP and PVA/borax of 100:1 possessed best mechanical properties due to the more inter-/intra-molecular hydrogen bonding compared to the PVA/borax hydrogel. High water content as well as the related properties made the PVP/PVA/borax hydrogel display a great potential for applications in wound dressing, bandage covering or sewage disposal and so on. It was no doubt that the current study would contribute to expand the future applications of PVA-based hydrogels.

## Disclosure statement

No potential conflict of interest was reported by the authors.

## Funding

The authors would like to appreciate the funding support of National Natural Science Foundation of China [grant number 51673131]; Project of Science and Technology Department of Sichuan Province [grant number 2016FZ0081]; Provincial Academic and Technical Leaders Foundation of Sichuan Province [grant number 2016-183-29].
